# Probable Loeys–Dietz Syndrome with aortic root dilatation and multiple arterial tortuosity in resource-limited setting : A case report

**DOI:** 10.1016/j.radcr.2026.09.066

**Published:** 2026-10-16

**Authors:** Nikita Datta, Zakia Akther Zahan, Nafisa Zaman Akhtar, Munim Hasan Choudhury, Amit Hasan, Rifah Anjum Samiha, Md. Deluwar Hussen

**Affiliations:** a Dhaka Medical College, Secretariat Road, Shahbagh, Dhaka, Bangladesh; b Dhaka Central International Medical College and Hospital, Shymoli Ring Road, Dhaka, 1207, Bangladesh; c Dinajpur Medical College, Dinajpur, Bangladesh; d Anwer Khan Modern Medical College Hospital, House: 17, Road: 08, Dhanmondi, Dhaka, 1205, Bangladesh; e Sir Salimullah Medical College, Mitford Road, Dhaka, 1100, Bangladesh

**Keywords:** Loeys-Dietz Syndrome, Connective tissue diseases hereditary, Aortic aneurysm, Aortic root dilatation, Computed tomography angiography, Case report

## Abstract

Loeys–Dietz Syndrome (LDS) is a rare hereditary connective tissue disorder that is characterized by multisystem disorders predisposing individuals to arterial and aortic aneurysms. We present the case of a 17-year-old Bangladeshi male referred to cardiology for assessment of systemic abnormalities and cardiovascular risk. He presented with craniofacial, musculoskeletal, and cutaneous abnormalities including hypertelorism, mandibular ectopic hair, joint hypermobility with mild thoracic scoliosis, residual talipes equinovarus. He had past medical history of surgically corrected congenital anomalies. Based on clinical and radiological findings, he was diagnosed with clinically suspected Loeys–Dietz Syndrome as genetic testing was unavailable. He was managed through a multidisciplinary approach with regular follow-up. This report adds to literature on Loeys–Dietz Syndrome, emphasizing on aortic root dilatation and carotid artery tortuosity as important clinical and radiological features supporting diagnosis of LDS particularly in young patients presenting with characteristic craniofacial abnormalities or suggestive radiological findings. Early recognition and multidisciplinary management are crucial in resource-limited settings to prevent life-threatening vascular complications.

## Introduction

Loeys–Dietz syndrome (LDS) is a rare autosomal dominant connective tissue disorder characterized by widespread arterial tortuosity, hypertelorism, and a bifid or broad uvula or cleft palate. The condition arises from mutations in transforming growth factor receptor genes like beta receptor-1 (TGFBR1) and beta receptor-2 (TGFBR2) [1]. It has an estimated prevalence of approximately 1 in 50,000 individuals [2]. First described in 2005, LDS may be diagnosed through radiological evaluation or confirmed genetically. Confirmatory diagnosis is established by identifying a heterozygous pathogenic variant in SMAD2, SMAD3, TGFB2, TGFB3, TGFBR1, or TGFBR2, or biallelic pathogenic variants in IPO8, particularly in the context of aortic root enlargement, type A dissection, or relevant family history [3]. Loeys–Dietz syndrome shares phenotypic features with other hereditary connective tissue disorders, making diagnosis based solely on clinical or radiological findings difficult [4]. Among its systemic manifestations, arterial vasculopathies tend to be the most aggressive and life-threatening component, where LDS carries the highest mortality among hereditary connective tissue disorders. We present a case of a 17-year-old male with multiple congenital anomalies in whom possible Loeys–Dietz syndrome was identified at an early stage. In our resource-limited clinical setting where advanced genetic testing remains readily inaccessible, early radiological diagnosis facilitated timely surveillance and proactive planning of interventions in the event of vascular complications. This case aims to aid clinicians in recognizing probable LDS without genetic confirmation, and it highlights the critical role of multidisciplinary surveillance to anticipate complications and reduce morbidity.

## Case presentation

A 17-year-old Bangladeshi male presented to the Medicine Unit, at a tertiary care hospital in Dhaka, Bangladesh with a constellation of symptoms suggestive of a connective tissue disorder. Those features included hypertelorism, ectopic hair along the lower mandibular border, bifid uvula, mildly prognathic jaw, joint hypermobility with mild thoracic scoliosis and residual talipes equinovarus deformity of the left foot. He was then referred to the Cardiology unit for assessment of systemic anomalies and cardiovascular risk with intention to screen for associated connective tissue disorder. His past medical history included left undescended testis, which was surgically corrected in early childhood, inguinal hernia, which was repaired at age 5 years and congenital clubfoot (talipes equinovarus), which was treated with serial casting and Achilles tenotomy. Slit lamp examination excluded ectopia lentis. Cognitive function was normal and there was no history personal history of cardiovascular or cerebrovascular events. His developmental history was notable for delayed motor milestones and persistent joint laxity. There was no known family history of aortic disease, connective tissue disorder, sudden cardiac death or early onset stroke. Socially, he was a student from a middle-class urban family, non-smoker, and abstinent from alcohol or illicit substances.

On general examination, the patient was thin and small for his age, with a weight of 28.3 kg and a height of 133 cm (BMI 15.9 kg/m^2^). Characteristic craniofacial features were observed, including hypertelorism, ectopic hair along the lower mandibular border, bifid uvula upon oral inspection and mildly prognathic jaw. Musculoskeletal assessment revealed generalized joint hypermobility (Beighton score 7/9), arachnodactyly, and mild pectus excavatum along with mild thoracic scoliosis. Residual talipes equinovarus deformity of the left foot was also observed. Skin examination showed normal texture and elasticity without significant translucency or easy bruising. Cardiovascular examination revealed a regular heart rate of 123 beats per minute and a Grade II/VI early diastolic murmur at the left upper sternal border, suggestive of aortic regurgitation.

### Differential diagnosis, investigations and treatment

Based on the clinical features of hypertelorism, bifid uvula, and aortic root dilatation, a clinical diagnosis of probable Loeys–Dietz Syndrome (LDS) was established, while genetic confirmation was pending. This conclusion is supported by the widespread arterial tortuosity observed on MRA, features which are significantly more pronounced in LDS than in related connective tissue disorders. This diagnosis is further favored by presence of characteristic craniofacial anomalies specifically bifid uvula, hypertelorism, ectopic mandibular hair and skeletal manifestations including joint hypermobility, scoliosis, talipes equinovarus. Although Marfan syndrome was considered due to aortic root dilatation, it was excluded based on the absence of ectopia lentis and the presence of a distinct craniofacial triad atypical of Marfan syndrome. Vascular Ehlers–Danlos syndrome was also considered given the arterial dilatation and fragility but was less likely in the absence of characteristic thin facial features and translucent skin. Despite strong clinical features supporting Loeys–Dietz syndrome, definitive genetic subtype confirmation was not possible due to the absence of genetic testing, the gold standard for differentiating aortopathies. (Fig. 1, Fig. 2, Fig. 3, Fig. 4, Fig. 5, Fig. 6)

**Fig. 1:**
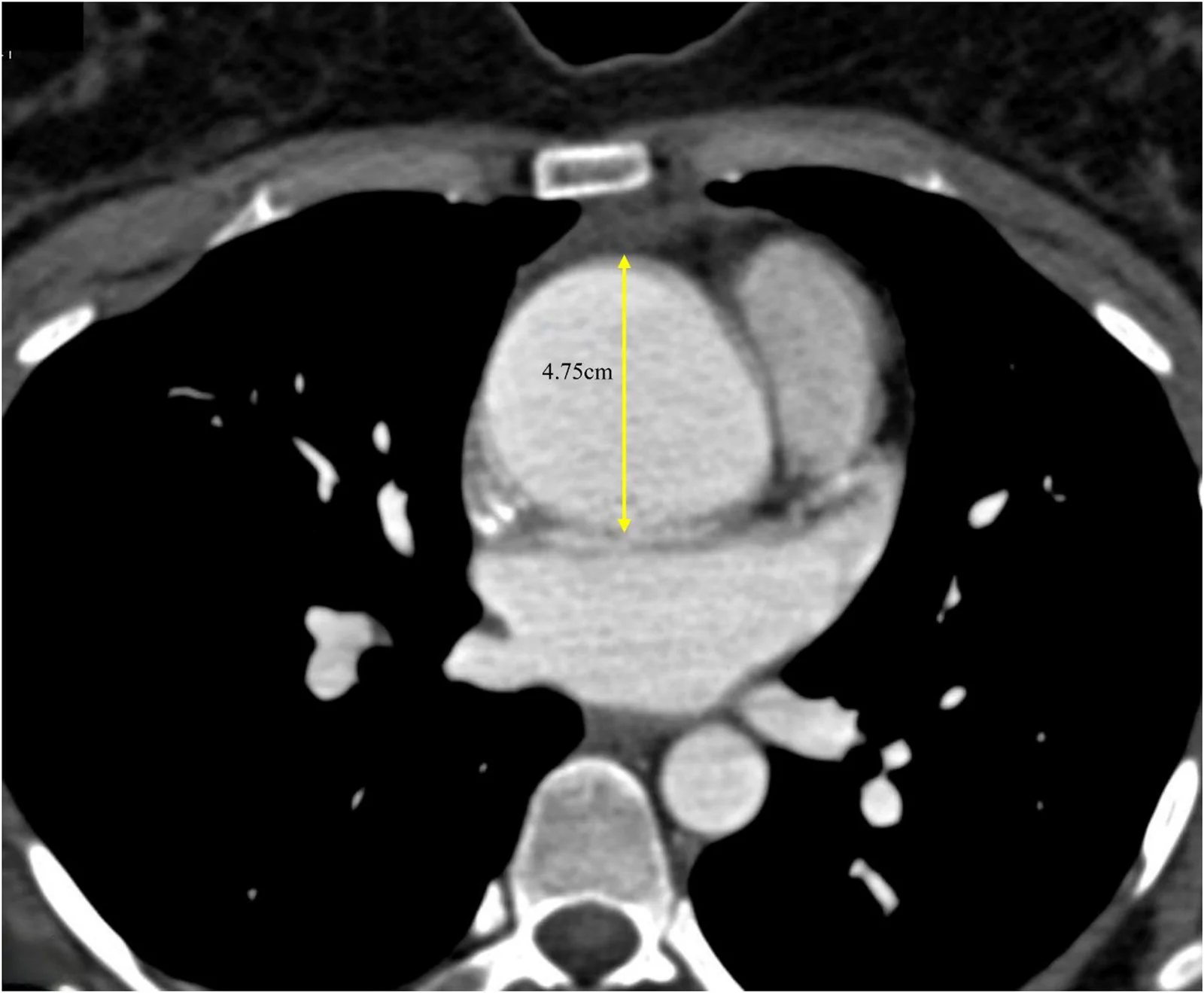
Axial contrast enhanced CT angiogram of the thorax demonstrates dilatation of the ascending aorta measuring 4.75 cm in diameter (yellow arrow), without evidence of dissection flap at the level of pulmonary artery bifurcation.

**Fig. 2:**
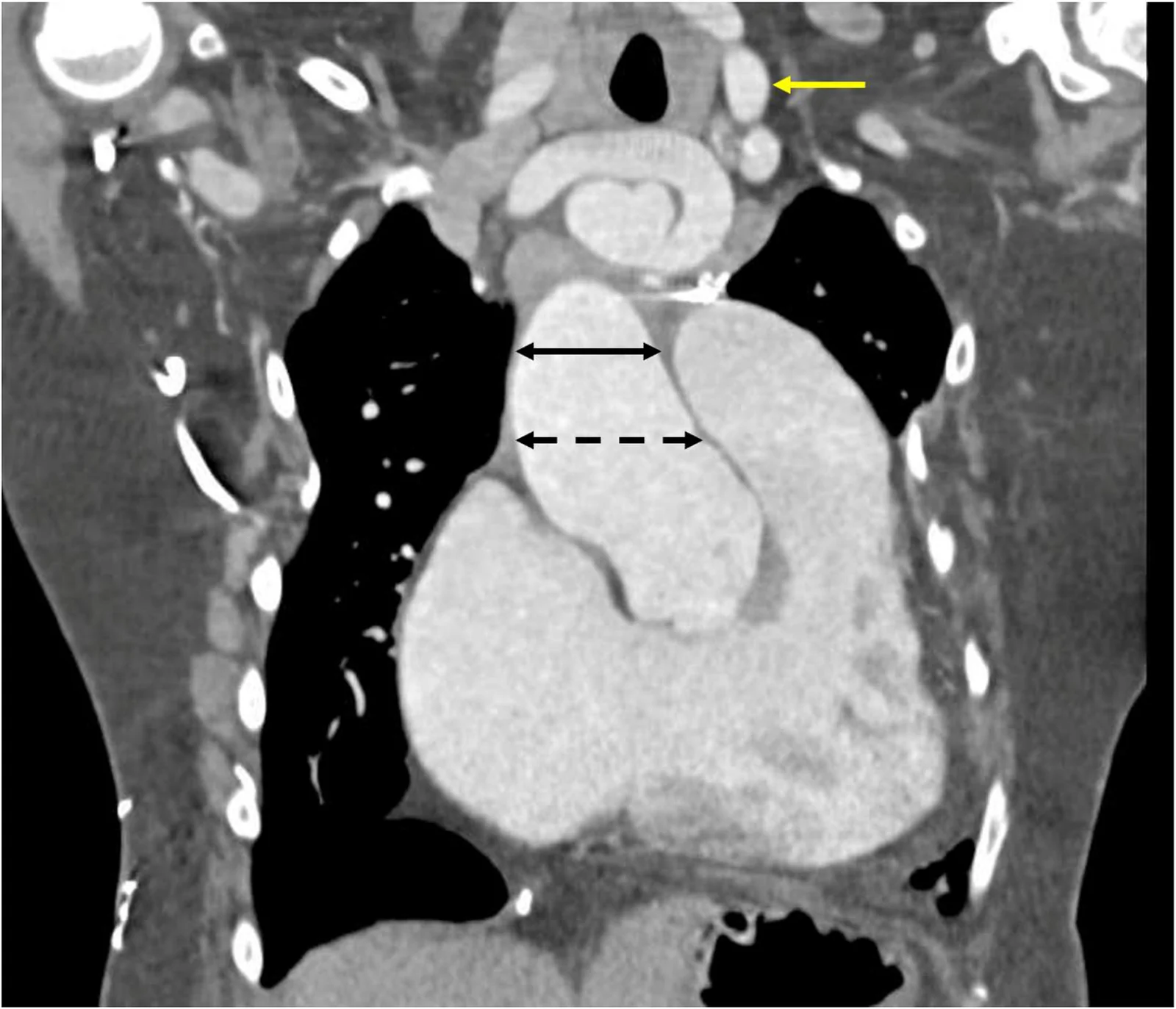
Coronal view of Contrast Enhanced CT Angiography Demonstrating Aortic Root (dashed black arrow) and Ascending Aortic (black solid arrow) Dilatation with associated arterial tortuosity (yellow arrow), suggestive of generalized aortic disease.

**Fig. 3:**
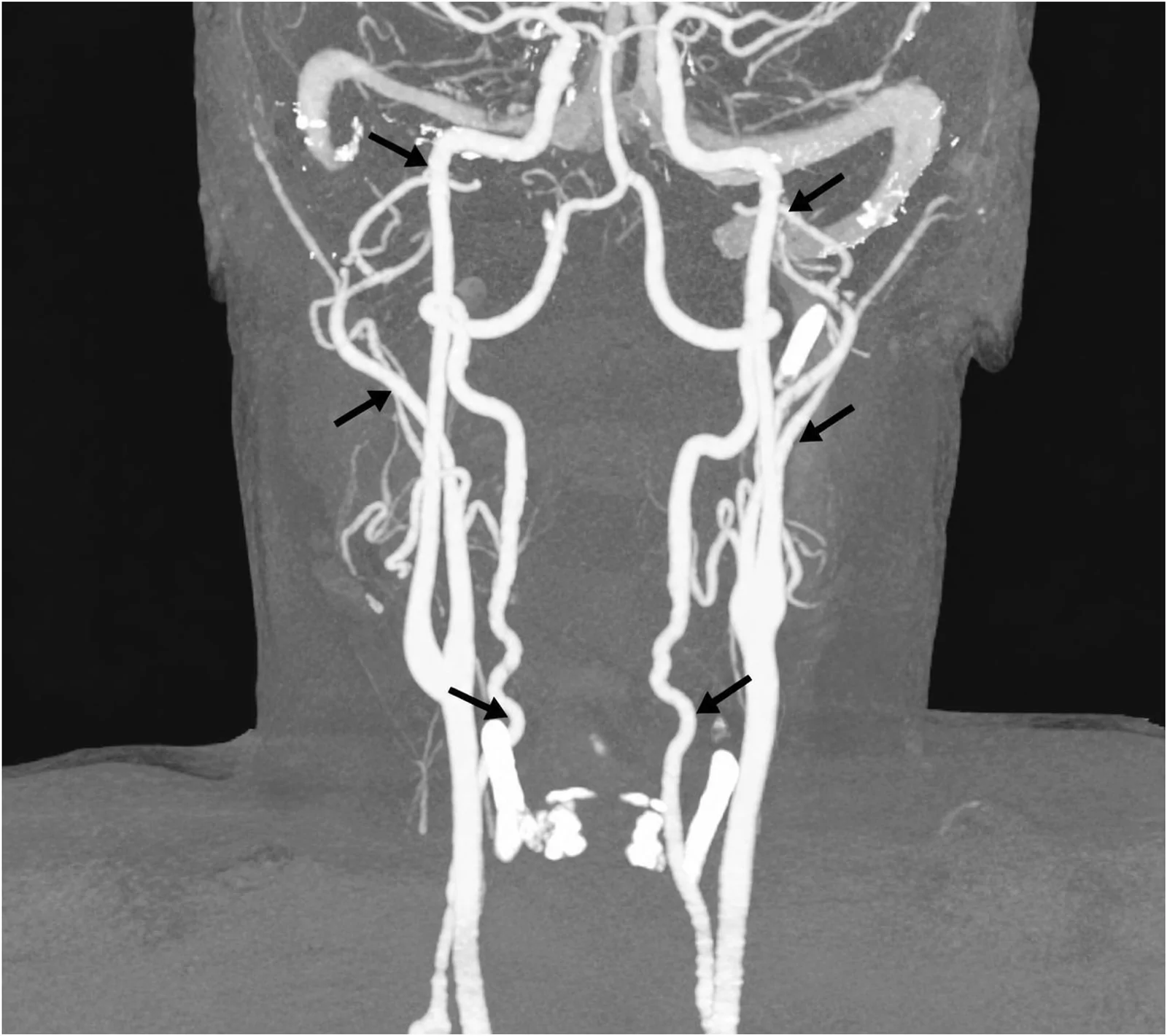
This 3D volume rendered reconstruction of a CT Angiogram showing marked tortuosity of bilateral carotid arteries and vertebral arteries marked by black arrows. Bone structures have been subtracted.

**Fig. 4:**
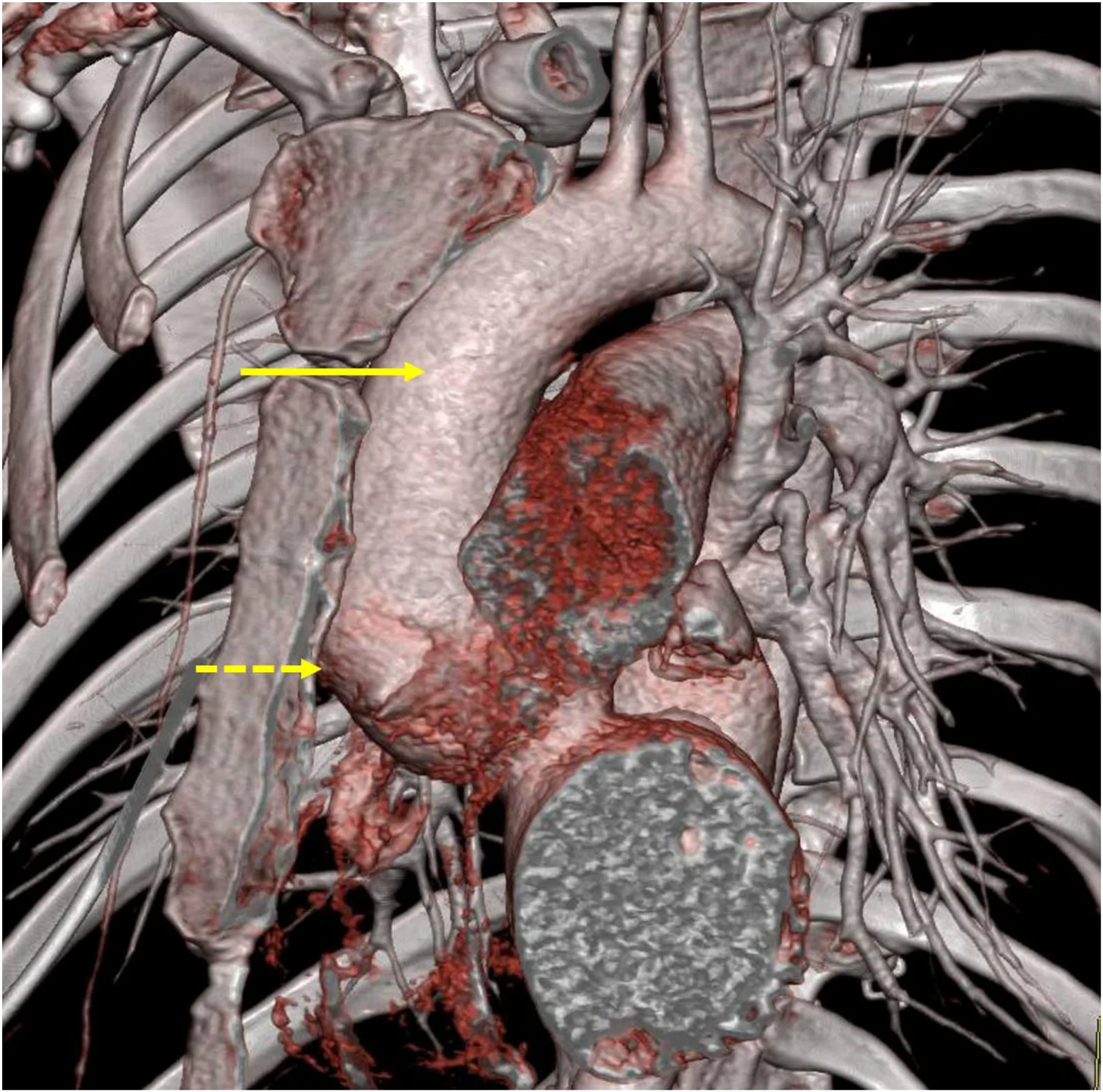
This Axial 3D volume rendering from IV Contrast enhanced CT angiography showing prominent dilatation of the aortic root (dashed yellow arrow) and ascending aorta (solid yellow arrow) within the chest cavity, confirming ascending aortic aneurysm with root involvement.

**Fig. 5:**
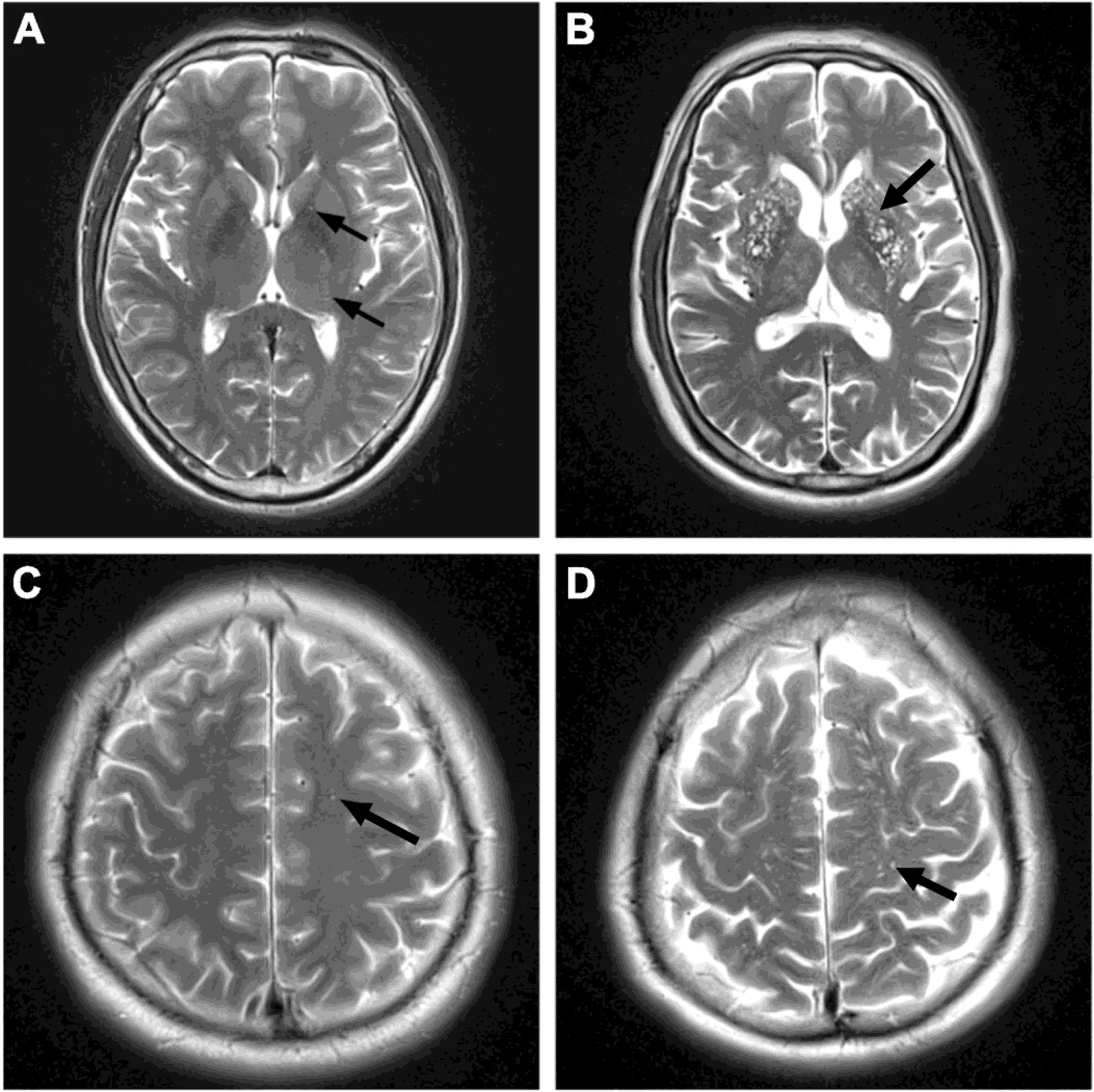
Axial T2-weighted brain MRI demonstrating multiple enlarged perivascular (Virchow–Robin) spaces at different anatomical levels: (A) Bilateral basal ganglia showing prominent perivascular spaces (black arrows); (B) Extensive, bilaterally symmetric, clustered perivascular spaces within the basal ganglia (black arrow); (C–D) Scattered punctate and linear hyperintense foci in the bilateral subcortical white matter (black arrows), consistent with enlarged perivascular spaces.

**Fig. 6:**
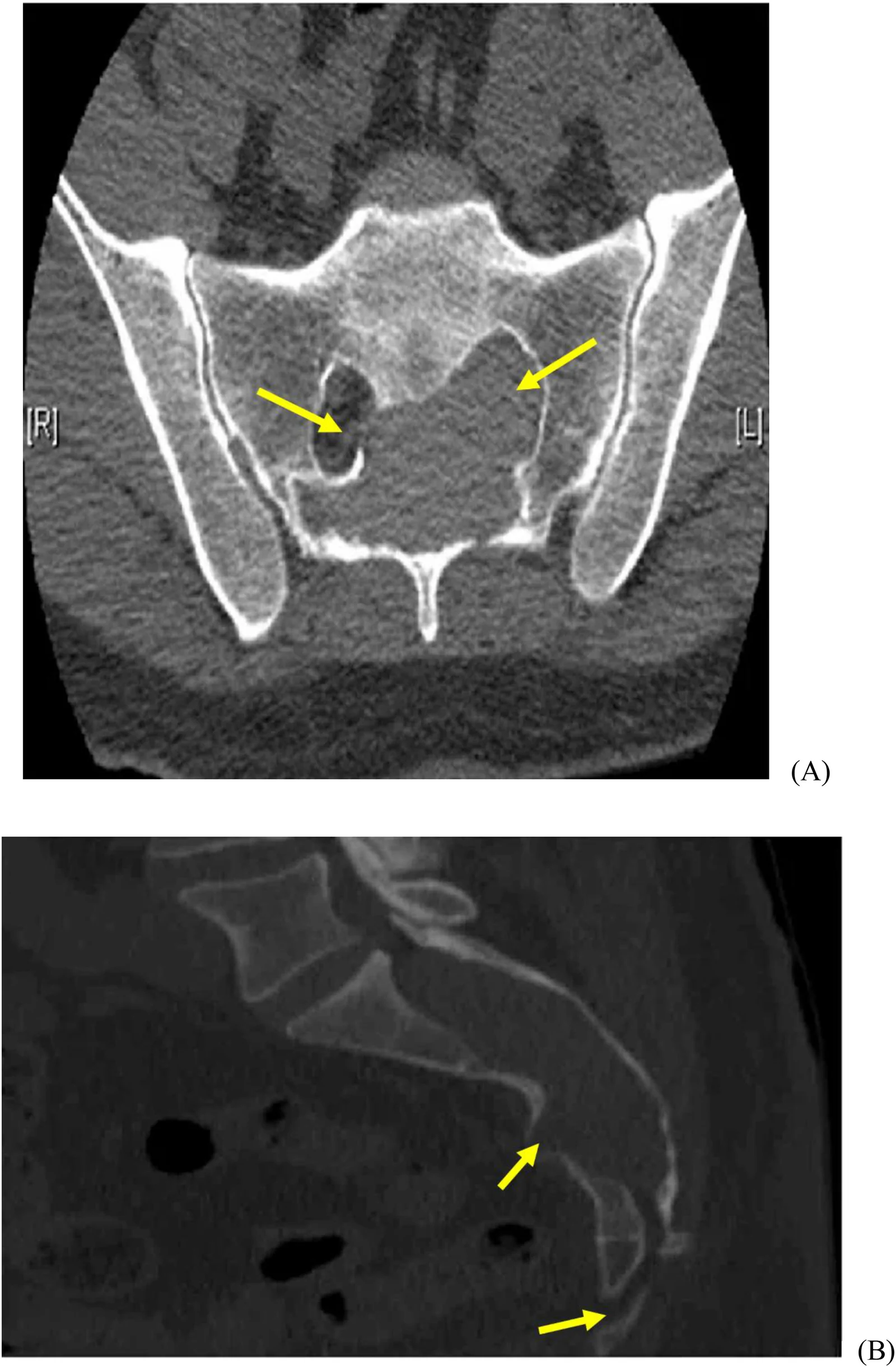
Axial (A) and sagittal (B) CT images of the pelvis demonstrating enlarged sacral foramina with smooth cortical margins (yellow arrows), suggestive of dural ectasia associated with an underlying connective tissue disorder.

Initial diagnostic imaging focused on assessment of the aortic anatomy. Contrast-enhanced CT of the chest and abdomen was performed using retrospective ECG-gated acquisition protocol, followed by transthoracic echocardiography (TTE), CT angiography (CTA), magnetic resonance angiography (MRA), and craniospinal imaging. Standardized multiplanar reformations (MPR) perpendicular to the axis of blood flow showed the following measurements alongside reference ranges in Table 1:

**Table 1:** Summary of cardiovascular, vascular, neurological, and spinal imaging findings.

Investigation	Findings
**CET aorta**	Dilated aortic root and ascending aorta, ascending aortic diameter at the level of the pulmonary artery bifurcation measured at **4.75 cm** in maximum outer diameter (leading edge to leading edge at maximal systole) **[**Fig. 1**]**
	**Aortic annulus was measured at 1.8 cm**
	**Aortic root at 2.99 cm** on echocardiographic end-diastolic inner-to-inner measurement; Z-score significantly >+4.0) [reference range: 1.6 cm to 2.2 cm]
	**Sinotubular junction at 3.8 cm**; Z-score >+4.0 [reference range: 1.3 cm to 1.8 cm]
	**Aortic arch** was mildly prominent, measuring **2.2 cm [reference range: 1.2 cm to 1.7 cm]**
	**Descending thoracic aorta was 2.01 cm** at the diaphragmatic level; posteriorly displaced [reference range: 1.0 cm to 1.4 cm]
	**Patent ductus arteriosus was** hemodynamically insignificant
**CTA/MPR [Coronal view]**	Confirmed aortic root and ascending aortic dilatation [Fig. 2].
**3D reconstruction CTA**	Prominent widening of the aortic root and ascending aorta **[**Fig. 4], with diffuse tortuosity of the bilateral carotid and vertebral arteries [Fig. 3].
**Cerebral and cervical MRA**	Marked tortuosity of bilateral carotid, vertebral, and middle cerebral arteries, greater on the left. No saccular or branch-vessel aneurysm or arterial dissection.
**Cranial MRI**	Bilateral punctate lesions at the level of basal ganglia, consistent with enlarged perivascular spaces; with no associated white-matter abnormality, restricted diffusion, or hemorrhage [Fig. 5].
**Spinal/pelvic imaging [Axial and Sagittal view]**	Bilaterally enlarged sacral neural foramina with smooth, well-corticated margins, consistent with dural ectasia, related to underlying connective tissue dysplasia connective tissue dysplasia; no aggressive bone destruction or cortical erosion [Fig. 6].
**TTE**	Dilated aortic root with preserved left ventricular function and no valvular disease.

Z-scores were calculated using standard pediatric and adolescent aortic dimension references indexed to the patient's body surface area (1.02 m^2^, calculated via the Mosteller formula). It revealed a value significantly exceeding +4.0, which confirmed moderate to severe dilatation across the root and ascending segments.

The patient was started on aggressive medical stabilization and lifestyle modifications to mitigate the risk of aortic dissection and rupture. Angiotensin II receptor blocker Losartan was initiated at 12.5 mg once daily (based on his weight of 28.3 kg, starting at ∼ 0.4 mg/kg). The dose was increased every 2–4 weeks to a target dose of 25 mg to 50 mg daily (∼0.8–1.7 mg/kg) to antagonize the overactive TGF-β signaling pathway and to maintain the systolic blood pressure adequately for perfusion. Alongside with Losartan, Beta Blocker Atenolol was initiated at a starting dose of 12.5 mg daily, which was titrated to achieve a resting heart rate of 60–70 bpm for the reduction of hemodynamic shear stress. As the patient’s stature was small, close monitoring of blood pressure and renal function checks along with serum electrolytes were ensured.

As vascular fragility in LDS requires strict physical restrictions to prevent acute dissection or rupture, high intensity isometric exercises or contact sports were strictly prohibited. Low impact aerobic exercises such as walking, light cycling or swimming were advised as long as the patient can maintain conversation while exercising.

### Outcome and follow-up

Because LDS is a more aggressive vasculopathy than Marfan Syndrome and Ehlers–Danlos Syndrome, surveillance was performed frequently. Follow up Echocardiography was scheduled at every 6 months to monitor the aortic root diameter. On transthoracic echocardiography, the aortic root (sinus of Valsalva) was measured 2.85 cm (inner edge to inner edge diameter, end diastole). The apparent decrease therefore reflects differences in the anatomic site, imaging modality and measurement convention rather than a true interval reduction in aortic dimensions. Additional recommendations were provided to the patient which included repeated cervical and intracranial MRA in every 12 months to screen for new aneurysms. Genetic counseling with cascade testing for first degree relatives, referrals to orthopedics for scoliosis management and dermatology for skin assessment were done. Considering his small BSA, the initial diameter of ascending aorta of 4.75 cm, aortic root of 2.99 cm and follow up diameter of aortic root of 2.85 cm, both values corresponded to a Z -score in the moderate to severe dilatation range and he remained in the medical management phase. Surgical referral criteria were discussed with the family. We carefully followed up with the patient for any of the following ascending aortic diameter approaching 4.0–4.4 cm (given the lower rupture threshold in LDS), a Z-score >4 with progressive increase, new aortic regurgitation, or rapid expansion (>0.5 cm in a year). The patient was carefully followed up against these thresholds to determine timing of any future surgical consultation.

At the 6-month follow-up, the patient remained asymptomatic with stable cardiovascular imaging findings. He continues regular multidisciplinary follow up until now with a coordinated care plan aimed at early detection of vascular complications and timely surgical intervention.

## Discussion

Loeys–Dietz syndrome (LDS) is an autosomal dominant connective tissue disorder that has been associated with extensive systemic involvement including craniofacial, skeletal, cutaneous, and vascular (arterial tortuosity, aneurysm formation, and dissection) abnormalities [5].Hallmark findings of LDS are early and aggressive aortic and arterial aneurysms and dissection [2].

LDS is a recently identified connective tissue disorder that shares clinical overlap with both Marfan Syndrome (MFS) and Ehlers–Danlos syndrome (EDS). However, certain differentiating features help to distinguish these diseases. For example, ectopia lentis is a distinguishing feature of MFS, whereas hypertelorism and bifid uvula are characteristic of LDS and are not seen in MFS [5]. In our patient, Ectopia lentis was excluded on slit-lamp examination. The vascular risks in LDS can be more severe than those in Marfan Syndrome (MFS), as aortic dissection has been reported at root diameter 3.9–4.0 cm which is significantly smaller than the 5.0 cm cutoff typically used as a surgical threshold in MFS [1]. Similarly, EDS can be diagnosed when a patient has at least two of these features: thin translucent skin, arterial, intestinal or uterine rupture, easy bruising and characteristic facial appearance. These features are absent in LDS [6]. In this case, the patient has hypertelorism and a bifid uvula which are considered as characteristic and often diagnostic features of LDS. LDS can be distinguished from Ehlers–Danlos syndrome (EDS) by its relatively better surgical outcomes, attributed to less tissue fragility and consequently lower intraoperative mortality [7]. That is why, early phenotypic recognition and accurate imaging interpretation are highly important for appropriate management and differentiation from conditions like MFS and EDS [8].

Heterozygous mutations in 6 genes (TGFBR1/2, TGFB2/3, SMAD2/3), which encodes components of the TGF-β pathway, cause LDS [9]. While the traits of classic *TGFBR1* and *TGFBR2* LDS are well established, newer variants (*TGFB2, TGFB3, SMAD2, SMAD3*) require further study because these newer patients often experience milder cardiovascular issues and lower genetic penetrance. Therefore, developing gene-specific vascular management strategies instead of relying on a uniform treatment model for all LDS patients is a clinical necessity [10]. However, clinicians working at peripheral medical college hospitals in remote areas like us often face limitations in diagnosing LDS due to limited resources and patients’ financial inability to afford diagnostic investigations like genetic testing. In that case, a clinical diagnosis is established based on the combination of cardiovascular abnormalities, characteristic phenotypic manifestations and imaging findings from modalities such as CTA or MRA.

To assess aortic root dilation in Marfan, Loeys–Dietz and Ehlers–Danlos syndromes, cardiologists use nomograms to calculate a z-score adjusted for age, sex and BSA, where an aortic root z-score ≥ 2 confirms pathological dilation [11]. In our patient, z-score > +4.0 as well as contrast CT and echocardiography of the patient confirmed moderate-to-severe dilatation across both the aortic root and ascending aorta, measuring 3.8 cm at the sinotubular junction and up to 4.75 cm in maximal outer diameter along with mild enlargement in the aortic arch (2.2 cm) and descending aorta (2.01 cm), which coincide with the aggressive aortic findings of LDS.

Surgical thresholds differ across the three conditions and are increasingly genotype-guided. The 2022 ACC/AHA guideline on aortic disease recommends prophylactic aortic root replacement in Marfan syndrome at a diameter of 4.5–5.0 cm, whereas LDS carries a lower threshold of 4.0–4.5 cm depending on the specific gene involved. In vascular Ehlers–Danlos syndrome(vEDS), prophylactic aortic root repair is largely avoided because of the high risk of intraoperative and anastomotic complications from tissue fragility [12]. Surveillance strategy also differs: Marfan syndrome surveillance is centered on serial echocardiography of the aortic root while LDS requires head-to-pelvis vascular imaging given its diffuse arteriopathy and risk of aneurysm formation at unpredictable sites [13]. Selecting an imaging modality for surveillance is a critical clinical decision. Because LDS demands lifelong monitoring often starting in childhood. MRA is preferred for routine check-ups to avoid cumulative radiation. Meanwhile, CTA's superior spatial resolution makes it the top choice for mapping highly tortuous vessels, establishing baseline evaluations and managing acute emergencies [1].

Medical therapy frequently fails to stop fast aortic expansion on its own, with 17% of extra-aortic aneurysms expanding over time and 38% of those expansions causing severe events like rupture or dissection. Consequently, routine annual check-ups using aortic root echocardiograms alongside head and neck CTA or MRA are essential to track vascular growth and time preventative surgeries before life-threatening complications occur [14]. In addition to, in LDS, the disrupted TGF-β pathway causes kidney fibrosis and progressive damage. Hardened arteries also cause common high blood pressure which accelerates this renal decline if left unchecked [1]. Therefore, checking blood pressure, serum creatinine and urine protein serves as the baseline monitoring standard. In this case, the patient is currently managed through regular multidisciplinary follow up with close cardiovascular surveillance and preventive strategies aimed at early detection of disease progression and timely surgical intervention.

## Conclusion

Aortic root dilation together with carotid artery tortuosity should raise immediate suspicion for LDS prompting rapid genetic confirmation and comprehensive vascular assessment. However, clinicians working at peripheral medical college hospitals in remote areas like ours often face challenges in diagnosing conditions such as LDS due to limited resources, financial constraints of patients and lack of access to genetic confirmation and advanced vascular imaging.

## Author contributions

1. **Dr. Nikita Datta, MBBS (Dhaka Medical College, Dhaka):** Dr. Nikita performed the literature review and drafted the manuscript.
2. **Dr. Zakia Akther Zahan, MBBS (Dhaka Central International Medical College and Hospital, Dhaka):** Dr. Zakia performed the literature review and revised the manuscript for important intellectual content.
3. **Dr. Nafisa Zaman Akhtar, MBBS (Dinajpur Medical College, Dinajpur):** Dr. Nafisa performed the literature review and revised the manuscript for important intellectual content.
4. **Dr. Munim Hasan Choudhury, MBBS (Anwer Khan Modern Medical College Hospital, Dhaka):** Dr. Munim contributed to figures formatting, manuscript editing, critical revision for intellectual content and language refining.
5. **Amit Hasan, MBBS (Sir Salimullah Medical College, Dhaka)**: Amit Hasan revised the manuscript for important intellectual content.
6. **Dr. Rifah Anjum Samiha, MBBS (Anwer Khan Modern Medical College Hospital, Dhaka):** Dr. Rifah contributed to, critical revision for intellectual content and proofreading.
7. **Dr. Md. Deluwar Hussen, MBBS (Dinajpur Medical College, Dinajpur**): Dr. Deluwar was involved in the formulation of the study, as well as the attainment of the data analysis and evaluation for the study. He also contributed to the writing of the review of literature and the manuscript and the review and editing of the manuscript.

All authors read and approved the final manuscript.

## Patient consent

Informed written informed consent was obtained from the patient for publication of the clinical details and accompanying images in this case report.
